# Bailout for the Difficult Gallbladder: Subtotal vs. Open Cholecystectomy—A Retrospective Tertiary Care Center Experience

**DOI:** 10.3390/medicina60101642

**Published:** 2024-10-08

**Authors:** Abdullah Aloraini, Ahmed Alburakan, Fatimah Saad Alhelal, Ghada Alabdi, Hend Elmutawi, Najd Saeed Alzahrani, Sarah Alkhalife, Tariq Alanezi

**Affiliations:** 1 Division of General Surgery, Department of Surgery, College of Medicine, King Saud University, Riyadh 11322, Saudi Arabia; abdaloraini@ksu.edu.sa; 2 Trauma and Acute Care Surgery Unit, Department of Surgery, College of Medicine, King Saud University, Riyadh 11322, Saudi Arabia; aalburakan@ksu.edu.sa; 3 College of Medicine, King Saud University, Riyadh 11322, Saudi Arabia; fatimah.alh22@gmail.com (F.S.A.); ghadaalabdi19@gmail.com (G.A.); halmotywea@gmail.com (H.E.); alzahrani.najd@gmail.com (N.S.A.); sarahalkhalife@gmail.com (S.A.)

**Keywords:** cholecystectomy, subtotal, laparoscopic cholecystectomy, open cholecystectomy, difficult gallbladder, bailout

## Abstract

*Background and Objectives:* A difficult gallbladder anatomy augments the risk of bile duct injuries (BDIs) and other complications during a laparoscopic cholecystectomy. This study compares the outcomes of a laparoscopic subtotal cholecystectomy (LSTC) and open total cholecystectomy (OTC) for difficult cholecystectomies. *Materials and Methods:* This retrospective analysis of gallbladder procedures (LSTC or OTC) from 2016 to 2023 examined patient demographics, surgical details, and postoperative results. The primary outcome was the incidence of a BDI. Secondary outcomes included operative duration, blood loss, and postoperative complications. *Results:* Seventy-one patients were included in the study. Of them, 59.2% (n = 42) underwent an LSTC and 44.6% (n = 29) underwent an OTC. The LSTC cohort was more likely to have a day-surgery case with a same-day discharge (33.3% vs. 0%, *p* = 0.009), less blood loss (71.4 ± 82.26 vs. 184.8 ± 234.86, *p* = 0.009), and a shorter operative duration (187.86 ± 68.74 vs. 258.62 ± 134.52 min, *p* = 0.008). Furthermore, BDI was significantly lower in the LSTC group (2.4% vs. 17.2%, *p* = 0.045). However, there were no significant differences between the two groups concerning intraoperative drain placement, peri-cholecystic fluid collection, bile leak, and other complications (*p* > 0.05). *Conclusions*: LSTC is a safe and effective alternative to OTC for challenging gallbladder cases. Further studies with larger sample sizes and longer follow-up periods as well as different study designs are warranted.

## 1. Introduction

The advent of the laparoscopic cholecystectomy (LC) has revolutionized the surgical management of gallbladder diseases due to its minimally invasive nature. LC has become the gold standard for treating symptomatic cholelithiasis due to its advantages over open cholecystectomy, such as reduced postoperative pain, shorter hospital stays, and improved quality of life [1,2]. However, despite these advancements, managing a technically challenging subset of the patients, typically termed “difficult gallbladders”, remains a significant challenge. A “difficult gallbladder” encompasses a broad spectrum of complexities, including acute and chronic inflammation, fibrosis, adhesions, anatomical variations, gangrene, and Mirizzi Syndrome [3] (Table 1).

Therefore, patients with difficult gallbladders who undergo LCs face heightened risks of complications, such as bile duct injury (BDI), bleeding, conversion to open cholecystectomy, and subpar postoperative outcomes [4]. The burden of a difficult gallbladder is further exacerbated by the rising prevalence of obesity and the aging population, both of which are associated with an increased incidence of gallbladder disease [5]. Current figures estimate that 16% of LC patients present with a difficult gallbladder [6].

Choosing the optimal surgical approach for complex gallbladder conditions is vital for optimizing patient outcomes during and after surgery. It has been shown that difficult gallbladder conditions, such as adhesion at Calot’s triangle or fibrosis, are associated with an increased risk of conversion and BDIs [7,8,9]. The standard of care for a difficult gallbladder has historically been the conversion to an open total cholecystectomy (OTC) [10,11]. However, OTC is associated with increased postoperative pain, longer hospital stays, slower recovery, and higher rates of postoperative morbidity and mortality [12].

Given the potential risks associated with both LCs and OTCs in the context of a difficult gallbladder, alternative surgical strategies have been explored. One such approach is a subtotal cholecystectomy, which involves the removal of the gallbladder while leaving a portion of the posterior wall or the infundibulum in place to minimize the risk of a BDI [13]. A subtotal cholecystectomy can be performed using either laparoscopic (laparoscopic subtotal cholecystectomy (LSTC)) or open techniques [14]. Importantly, LSTC has emerged as a promising alternative to OTC in managing difficult gallbladders, offering the benefits of a minimally invasive approach while potentially reducing the risk of a BDI [15,16]. The utilization of LSTCs has significantly increased since its introduction in the early 1990s [16,17]. Previous reports showed that LSTCs were associated with lower risks of BDIs compared to a conversion to an OTC [18]. Nonetheless, LSTCs can increase the risk of bile leaks and postoperative complications due to the remnant gallbladder [19,20].

Recently, a number of studies compared the outcomes of LSTCs and the conversion to OTC for a difficult gallbladder, with conflicting results [11,21,22]. Hence, the comparative outcomes of LSTCs and OTCs in the context of a difficult gallbladder have not been well-established, necessitating further investigation. This retrospective study evaluated and compared the intra- and postoperative outcomes of LSTCs and conversion to OTCs in patients with difficult gallbladders at a single tertiary care center. Our research hypothesis posits that LSTC represents a safer and more effective alternative to OTC for managing difficult gallbladder cases. Specifically, we hypothesize that an LSTC results in lower rates of BDIs, reduced blood loss, and shorter operative durations compared to OTCs.

## 2. Materials and Methods

The study protocol was approved by the Institutional Review Board (IRB) of the College of Medicine, King Saud University, Reference No. E-24-8663 (16 April 2024). Given the retrospective nature of the study, the requirement for informed consent was waived by the IRB committee. The STrengthening the Reporting of OBservational studies in Epidemiology (STROBE) checklist was implemented [23].

### 2.1. Study Design and Population

This retrospective single-center study was conducted at King Khalid University Hospital. The medical records of patients who underwent either an LSTC or OTC for a difficult gallbladder between January 2016 and June 2023 were reviewed. The inclusion criteria were: (1) patients aged 18 years or older; (2) a preoperative diagnosis of symptomatic cholelithiasis, acute cholecystitis, or chronic cholecystitis; and (3) intraoperative documentation of difficult gallbladder conditions, including cases of acute cholecystitis with thick-walled, necrotic, or gangrenous gallbladders; chronic cholecystitis, where Calot’s triangle was obscured due to adhesions with a thick and contracted gallbladder; Mirizzi Syndrome; and suspected or known gallbladder perforation [3]. Patients were excluded from the study if they: (1) had a history of a previous cholecystectomy or if they were undergoing the completion a cholecystectomy, (2) had malignant gallbladder disease, (3) underwent concomitant biliary or hepatic resections, or (4) had incomplete medical records or insufficient follow-up data.

### 2.2. Data Collection

The extracted data from the medical records of eligible patients included preoperative data, such as age, sex, body mass index (BMI), the American Society of Anesthesiologists (ASA) physical status classification, smoking status, comorbidities, history of bariatric surgery, previous abdominal scaring, history of biliary inflammation (cholecystitis or cholangitis), cholecystitis type, operative priority, radiological findings, and laboratory findings. The severity of cholecystitis was classified according to the Tokyo Guidelines 2018 (TG18) grading [24]. We also studied the difference in the Randhawa scoring group score [25] and Tongyoo scoring group scores [26] between the two groups. The scores for were classified using previously reported groupings, i.e., “1–5 = easy”, “6–10 = difficult”, and “11–15 = very difficult.” The operative difficulty was graded as easy, difficult, and very difficult according to the criteria presented in Table 2. The degree of gallbladder wall thickness was classified as normal (1–2 mm), mildly thickened (3–4 mm), moderately thickened (5–6 mm), and severely thickened (≥7 mm) [27]. Concerning the intraoperative data, we collected operative duration, blood loss, BDI, and intraoperative drain placement. Postoperatively, we collected postoperative complications, including bile leak, peri-cholecystic fluid collection, and mortality, among others.

### 2.3. Statistical Analysis

The statistical analysis was conducted using R software for Windows (R version 4.3.1, 16 June 2023). Continuous variables were summarized using mean and standard deviation and compared between the two groups (laparoscopic and open) using a t-test or Wilcoxon’s test. Categorical variables were summarized using frequency and proportions and compared between the two groups using chi-squared or Fischer’s exact tests. A *p*-value of 0.05 or lower was considered statistically significant.

## 3. Results

A total of 3538 LC patient charts was reviewed. Of them, 71 patients were included in the present study with a bailout rate of 2.0%. Nearly 59.2% (n = 42) of the patients underwent LSTCs and 44.6% (n = 29) underwent OTCs. The mean age of the cohort was 50.3 ± 17.1 years, and 59.2% (n = 42) of the cohort were females. Additionally, the mean BMI of the included patients was 29.5 ± 6.9 Kg/m^2^, and six patients (8.5%) were smokers. The most common comorbidities were diabetes (31%) and hypertension (26.8%). We found no significant differences between the two groups in terms of age (*p* = 0.37), sex (*p* = 0.57), BMI (*p* = 0.18), smoker status (n = 64), or comorbidity status (all *p* > 0.05). However, patients in the OTC group were more likely to undergo an elective procedure (75.9%) compared to those in the LTSC group (45.2%, *p* = 0.010). Concerning the type, the majority of the patients had chronic cholecystitis (40.8%), followed by acute cholecystitis (31%). According to the TG18 criteria, most patients (78.6%) were grade 2, followed by 12 patients in the grade 1 category and four patients in the grade 3 category. There were no significant differences between the two groups with regard to the type or grade of cholecystitis (Table 3).

There was no statistically significant association between the type of surgical procedure and the presence of a radiologically contracted gallbladder (*p* = 0.202). Among patients who underwent LSTCs, 33.3% had a radiologically contracted gallbladder, compared to 21.4% in the OTC group. Similarly, there was no statistically significant association between the type of surgical procedure and the presence of an irregular or absent gallbladder wall (*p* = 0.659) and clinical palpability of the gallbladder (*p* = 0.262). The results show that patients in the LSTC group are more likely to have moderate or severe wall thicknesses (52.4%, n = 22) compared to patients in the open group (35.7%, n = 10) (*p* = 0.030). Upon comparing the laboratory resukts between the two groups, patients in the OTC had a significantly higher preoperative white blood cell (WBC) count (mean 11.14 ± 5.51 vs. 7.42 ±2.81) compared to the LSTC group (*p* = 0.002, Table 4).

**Figure 1:**
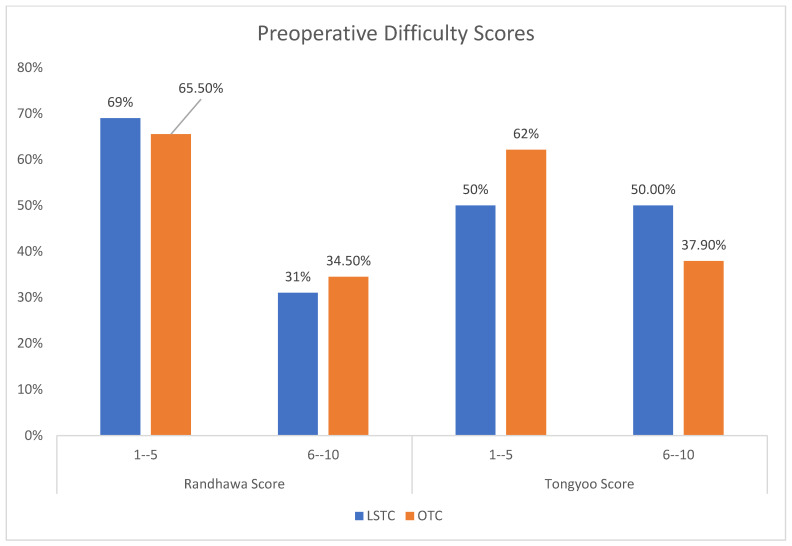
Preoperative difficulty scores. LSTC: laparoscopic subtotal cholecystectomy; OTC: open total cholecystectomy.

Regarding the preoperative assessment of preoperative difficulty, the results show no statistically significant association between the Randhawa scoring groups and the type of surgical procedure (*p* = 0.560). Nearly 31% of LSTC patients and 34.5% of OTC patients had a Randhawa score in the range of 6–10. In the case of the Tongyoo scoring groups, there was also no statistically significant association with the type of surgical procedure (*p* = 0.331). Half of the LSTC patients and 37.9% of OTC patients had a Tongyoo score in the range of 6–10 (Figure 1).

Regarding operative difficulty, there was no statistically significant difference between both groups (*p* = 0.052). In the LSTC group, 81% of the procedures was classified as very difficult, and 100% in the OTC group. Comparing the operative details, we found that patients in the LSTC group were more likely to have a day-surgery case with a same-day discharge (33.3% vs. 0%, *p* = 0.009). Patients in the LSTC group had significantly lower blood loss (74.4 ± 86.12 vs. 184.8 ± 234.87, *p* = 0.007) as well as a significantly shorter operative duration (183.86 ± 67.13 vs. 258.62 ± 135.52 min, *p* = 0.003). Patients in the LSTC group had a significantly lower incidence of BDI (2.4% vs. 17.2%, *p* = 0.027). On the other hand, there were no significant differences between the two groups in terms of intraoperative drain placement (*p* = 0.29), peri-cholecystic fluid collection (*p* = 0.94), bile leak (*p* = 0.31), and other complications (*p* = 0.091), Table 5.

## 4. Discussion

Managing difficult gallbladder conditions continues to be a challenge due to the heightened risk of BDIs and postoperative morbidity and mortality. Bailout LSTCs have emerged as a safe alternative to a conversion to OTCs that can significantly reduce the risks inherent to OTCs [30]. Although comparative studies have evaluated the outcomes of LSTCs and OTCs in difficult gallbladder cases, the published results are conflicting [14]. The present study compared the outcomes of LSTCs and OTCs in patients with difficult gallbladders. Our findings suggest that LSTC is a safe and effective alternative to OTC in managing difficult gallbladder disease, with several potential advantages. Our results demonstrate that the patients who underwent LSTCs had a significantly higher likelihood of having a day-surgery case with a same-day discharge, significantly lower blood loss, and significantly shorter operative duration compared to those who underwent OTCs. Additionally, patients in the LSTC group had a significantly lower incidence of BDIs compared to those in the OTC group. There were no significant differences between the two groups in terms of intraoperative drain placement, peri-cholecystic fluid collection, bile leak, and other complications.

BDIs are a significant burden for patients undergoing gallbladder surgery, as they can result in long-term morbidity and the need for complex reconstructive surgery, significantly impacting the patient’s quality of life [31]. Additionally, previous reports showed that BDIs can negatively impact long-term survival [32,33]. The risk of BDIs remains a major concern when dealing with difficult gallbladders, particularly in the presence of dense adhesions within Calot’s triangle, where a complex anatomy and inflammation increase the likelihood of misidentification and subsequent injury to the hepatic pedicle [30]. Since its introduction, LSTC has been advocated for reducing the risk of BDIs by partially removing the gallbladder wall while maintaining the advantages of the laparoscopic approach, such as reduced postoperative pain and total hospital stay [34,35]. In the present study, we found that the LSTC was associated with a lower risk of BDIs compared to OTCs. These findings run in line with a growing body of evidence showing a lower risk of BDIs in patients undergoing LSTCs. Recent meta-analyses showed a lower risk of BDIs with subtotal cholecystectomy compared to total cholecystectomy [14,36]. Other reports showed a low incidence of BDIs in patients undergoing LSTCs (0.08–0.18%) [30,37]. However, it should be noted that other recent studies showed a comparable rate of BDIs between bailout LSCTs and OTCs [38]. The same findings were observed by Grossman et al. [11] and Braschi et al. [39]. Still, even these results indicate that LSTC achieves its main objective of being comparable to a simple total cholecystectomy while maintaining the advantages of the laparoscopic approach [37]. The lower BDI rate observed in the LSTC group in our study may be attributed to several factors specific to the surgical technique, patient selection, and experience levels of the surgeons. Concerning surgical strategy, LSTC involves leaving part of the gallbladder intact, which avoids dissecting around Calot’s triangle, a region notorious for its complex and variable anatomy, particularly in difficult gallbladder cases. This reduces the likelihood of inadvertently injuring the bile duct during surgery. Moreover, LSTC allows surgeons to avoid dissecting dense adhesions or inflamed tissues around critical structures, which are common in difficult gallbladders. This conservative approach minimizes the need for aggressive manipulation, thereby reducing the risk of misidentifying or damaging the bile duct. Other key aspects can be highlighted. For example, the different outcomes in our study could reflect the expertise of surgeons familiar with this technique and skilled in determining when an LSTC is appropriate, especially in cases with severe inflammation or a complex anatomy. Our institute contains both skilled general surgeons and expert hepatobiliary surgeons. This expertise could have contributed to the lower BDI rates observed in the LSTC group compared to OTC.

Our analysis showed that LSTC was associated with lower blood loss, shorter operative duration, and a higher likelihood of having a same-day discharge. These findings align with previous reports demonstrating that the bailout LSTC was associated with a significantly shorter hospital stay, lower risk of intensive care admission, less bleeding requiring blood transfusions compared to OTC, and lower re-admission rates [11,38,39]. We also determined similar postoperative morbidity rates for LSTCs and OTCs, which tallies with several other retrospective studies [11,21,38,39,40]. In a previous systematic review, it was found that LSTC was associated with low rates of bleeding (0.54%), intra-abdominal collection (4%), wound infection (4%), and mortality (0.18%) [30], which are in line with our results. Nevertheless, inconsistencies in the literature should be acknowledged, as some studies point to a higher risk of postoperative morbidity with LSTCs. For instance, Koo et al. found that LSTC was associated with higher risks of postoperative endoscopic retrograde cholangiopancreatography, intraabdominal collections, and reoperation [14]. Thus, further studies with larger sample sizes and longer follow-up periods are warranted.

While LSTC has several advantages, it is important to acknowledge that it is not without drawbacks. One of the potential complications associated with LSTC is an increased risk of bile leak, with an incidence rate in the range of 10.6–18% [30,37]. The causes of bile leaks in LSTCs are multifactorial and complex. One possible factor is the presence of edematous tissue in the Hartmann pouch or cystic duct stump, which may become more susceptible to leakage as the edema subsides, leading to a loss of the watertight seal of the sutures. Furthermore, bile leakage may also occur from unsecured ducts, particularly if the cystic duct or gallbladder stumps are left open, allowing bile to accumulate in the subhepatic space [37,41]. In our experience, the rate of bile leak following an LSCT was not significantly different from OTC. Similarly, Grossman et al. showed no difference in the risk of bile leak between LSCT and OTC as bailout procedures [11]. On the contrary, Dominguez et al. [38] and Koo et al. [14] found that LSTC was associated with higher risks of bile leaks compared to OTC. Such heterogeneity in the published literature may stem from the differences in the employed LSTC techniques. It was previously suggested that bile leaks occur less in LSTCs when the stumps are securely closed [42,43]. Therefore, future studies are needed to compare directly between different LSTC techniques to investigate the impact of the LSTC technique on the risk of bile leaks.

The present study showed a single-center experience from a tertiary center in Saudi Arabia regarding the rate of bailout procedures and their outcomes. In our experience, the rate of bailout procedures was 2%; of them, 59.2% underwent LSTCs, indicating a general preference toward LSTCs in our center. The preference for performing LSTCs is likely attributed to the established practices of senior surgeons in the institute and the presence of a well-established hepatobiliary unit. Over the years, there has been a notable shift toward LSTCs. This trend may reflect the broader shift in surgical training toward the early introduction of laparoscopic and robotic approaches, resulting in a decline in experience with open surgery. As such, it is expected that the rate of OTCs will continue to decline as a newer generation of surgeons favors laparoscopic approaches when dealing with complex gallbladders [37,44].

### Study Limitations and Future Perspectives

Limited comparative studies evaluated the outcomes of LSTCs versus OTCs in patients with difficult gallbladders. However, we acknowledge that the present study has some limitations. The retrospective design of this study inherently carries certain biases and limitations, including missing or incomplete data, inconsistencies in the quality of documentation, recall bias, and potential misclassification bias. Furthermore, the retrospective data collection may have led to selection bias, as patients with specific characteristics or outcomes might have been more likely to be included in the study. The single-center design of the study restricts the generalizability of the findings to diverse patient populations. Lastly, the study did not categorize patients based on the severity of their gallbladder disease or previous treatment regimens. These factors could potentially influence the outcomes of the cholecystectomy procedures. Therefore, the lack of stratification might have introduced confounding variables, affecting the interpretation and generalizability of the study’s findings. Future research should consider these factors to provide a more comprehensive understanding of the comparative outcomes of LSTCs and OTCs.

Although our analysis is affected by these limitations, the results remain valuable and can contribute to the existing literature for several reasons. Specifically, despite being retrospective, our study captures real-world clinical outcomes over several years. This approach allows us to observe the natural course of LSTCs and OTCs in “difficult gallbladder” cases. It reflects a broad and diverse patient population. Additionally, since this study is one of the few to directly compare LSTC and OTC for difficult gallbladder cases, it provides novel insights into the perioperative outcomes, including operative duration, blood loss, and incidence of BDI, where LSTC demonstrated significant advantages. Furthermore, our findings support the growing trend of using LSTC as a safe alternative to OTC, particularly in complex cases where the risk of BDIs is higher. These results help strengthen the argument for laparoscopic approaches, even in challenging cases, aligning with current advancements in minimally invasive surgery. Given these results, the study paves the way for further research, particularly prospective studies, to confirm our findings and explore the long-term outcomes.

## 5. Conclusions

Findings from our retrospective analysis demonstrated that LSTC is a safe and effective alternative to OTC for patients with difficult gallbladder disease. Our results show that LSTC is associated with a significantly lower incidence of BDIs, shorter operative duration, and less blood loss when compared to OTC. These findings suggest that LSTCs may offer advantages over OTCs when managing difficult gallbladder disease, particularly in terms of the perioperative outcomes. Further studies with larger sample sizes are warranted to confirm these results and to better understand the long-term outcomes of LSTC compared to OTC.

## Figures and Tables

**Table 1:** Comparison of normal vs. difficult gallbladder anatomies.

Anatomical Feature	Normal Gallbladder	Difficult Gallbladder
Gallbladder Wall Thickness	1–2 mm (normal)	5–7 mm (moderately to severely thickened)
Calot’s Triangle Visibility	Visible	Obscured by adhesions, inflammation, or fibrosis
Adhesions	Absent	Present, often dense and fibrotic
Cystic Duct	Easily identifiable	Distorted or obscured due to inflammation
Gallbladder Perforation	None	Possible, especially in cases of necrosis or gangrene
Blood Supply (Cystic Artery)	Easily accessible and distinguishable	Compromised, requiring careful dissection
Bile Duct Proximity	No significant risk of bile duct injury (BDI)	Higher risk of BDI due to distorted anatomy

**Table 2:** Operative difficulty grading [26,28,29].

Grade	Parameters
Easy	Time taken: <60 min. No bile spillage. No injury to duct or artery
Difficult	Time taken: 60–120 min. Bile/stone spillage. Injury to the bile duct
Very difficult	Time taken: >120 min. Conversion

**Table 3:** Demographic and clinical characteristics of the patients.

	LSTC (N = 42)	OTC (N = 29)	Total (N = 71)	*p*-Value
Age, Mean (SD)	48.79 (15.57)	52.48 (19.13)	50.29 (17.08)	0.374
Sex				
Female	26 (61.9%)	16 (55.2%)	42 (59.2%)	0.571
Male	16 (38.1%)	13 (44.8%)	29 (40.8%)	
BMI, Mean (SD)	30.38 (6.381)	28.13 (7.45)	29.463 (6.88)	0.178
Smoker Status				0.638
Ex-Smoker	1 (2.4%)	0 (0.0%)	1 (1.4%)	
Non-Smoker	38 (90.5%)	26 (89.7%)	64 (90.1%)	
Smoker	3 (7.1%)	3 (10.3%)	6 (8.5%)	
Number of Comorbidities				0.811
0	20 (47.6%)	12 (41.4%)	32 (45.1%)	
1	10 (23.8%)	9 (31.0%)	19 (26.8%)	
2	3 (7.1%)	3 (10.3%)	6 (8.5%)	
3	6 (14.3%)	3 (10.3%)	9 (12.7%)	
4	3 (7.1%)	2 (6.9%)	5 (7.0%)	
HTN	12 (28.6%)	7 (24.1%)	19 (26.8%)	0.678
DM	15 (35.7%)	7 (24.1%)	22 (31.0%)	0.3
DLP	4 (9.5%)	3 (10.3%)	7 (9.9%)	0.909
CAD	0 (0.0%)	1 (3.4%)	1 (1.4%)	0.226
CVA	1 (2.4%)	0 (0.0%)	1 (1.4%)	0.403
Acute Pancreatitis	0 (0.0%)	1 (3.4%)	1 (1.4%)	0.226
Liver Disease	1 (2.4%)	1 (3.4%)	2 (2.8%)	0.789
Renal Insufficiency	2 (4.8%)	0 (0.0%)	2 (2.8%)	0.233
Other	8 (19.0%)	8 (27.6%)	16 (22.5%)	0.397
Previous Abdominal Surgical Scar	7 (16.7%)	4 (14.3%)	11 (15.7%)	0.789
Previous Bariatric Surgery	0 (0.0%)	1 (3.4%)	1 (1.4%)	0.262
Cholecystitis Type				0.232
Acute	15 (35.7%)	7 (24.1%)	22 (31.0%)	
Acute on Chronic	13 (31.0%)	6 (20.7%)	19 (26.8%)	
Chronic	14 (33.3%)	16 (51.7%)	29 (40.8%)	
Operative Priority				0.010
Elective	19 (45.2%)	22 (75.9%)	41 (57.7%)	
Emergency	23 (54.8%)	7 (24.1%)	30 (42.3%)	
ASA Group				0.968
1 or 2	39 (92.9%)	27 (93.1%)	66 (93.0%)	
3 or 4	3 (7.1%)	2 (6.9%)	5 (7.0%)	
Tokyo Guidelines Grading				0.143
Grade 1	10 (24.4%)	2 (6.9%)	12 (17.1%)	
Grade 2	29 (70.7%)	26 (89.7%)	55 (78.6%)	
Grade 3	3 (7.1%)	1 (3.44%)	4 (5.6%)	

ASA: American Society of Anaesthesiologists; BMI: Body Mass Index; CAD: Coronary Artery Disease; CVA: Cerebrovascular Accident; DLP: Dyslipidemia; DM: Diabetes Mellitus; HTN: Hypertension; LSTC: Laparoscopic Subtotal Cholecystectomy; OTC: Open Total Cholecystectomy; SD: Standard Deviation.

**Table 4:** Radiological and laboratory characteristics of the patients.

	LSTC (N = 42)	OTC (N = 29)	Total (N = 71)	*p*-Value
Radiologically contracted gallbladder? *	14 (33.3%)	6 (21.4%)	20 (28.2%)	0.202
Irregular or absent gallbladder wall? *	2 (4.8%)	2 (7.1%)	2 (2.8%)	0.659
Clinically palpable gallbladder?	0 (0.0%)	1 (3.4%)	1 (1.4%)	0.262
Gallbladder wall thickness (mm) classification				0.030
N-Miss	0	1	1	
Normal	9 (21.42%)	15 (53.6%)	22 (31.4%)	
Mild	10 (23.8%)	3 (10.7%)	12 (17.1%)	
Moderate	10 (23.8%)	3 (10.7%)	12 (17.1%)	
Severe	12 (28.6%)	7 (25%)	18 (25.71%)	
Total bilirubin, mean (SD)	12.958 (9.6)	16.926 (15.58)	14.626 (12.52)	0.323
Direct bilirubin, mean (SD)	8.208 (15.93)	9.863 (15.06)	8.970 (15.44)	0.469
ALP, mean (SD)	156.77 (161.27)	142.48 (88.58)	150.77 (134.89)	0.683
GGT, mean (SD)	141.45 (159.83)	118.52 (117.79)	131.81 (143.16)	0.365
WBC count, mean (SD)	7.42 (2.81)	11.14 (5.51)	9.42 (4.82)	0.002
Albumin level, mean (SD)	33.907 (7.13)	32.166 (6.96)	33.175 (7.06)	0.386
Platelet count, mean (SD)	305.60 (106.22)	279.76 (87.57)	294.74 (98.96)	0.244
INR, mean (SD)	1.06 (0.11)	1.03 (0.09)	1.05 (0.10)	0.263

* = Radiological details are missing for one patient in the open group. ALP: alkaline phosphatase; GGT: gamma-glutamyl transferase; INR: international normalized ratio; LSTC: laparoscopic subtotal cholecystectomy; OTC: open total cholecystectomy; SD: standard deviation; WBC: white blood cell count.

**Table 5:** Intra- and postoperative findings for the patients.

	LSTC (N = 42)	OTC (N = 29)	Total (N = 71)	*p*-Value
Operative Difficulty				0.052
Difficult	8 (19%)	0 (0.0%)	8 (11.3%)	
Very difficult	34 (81%)	29 (100%)	63 (88.7%)	
Day-surgery case	14 (33.3%)	0 (0.0%)	14 (19.7%)	0.009
Operative duration (min), mean (SD)	183.86 (67.13)	258.62 (135.52)	214.39 (106.57)	0.003
Blood Loss (ml), mean (SD)	74.41 (86.12)	184.83 (234.87)	119.51 (171.45)	0.007
BDI	1 (2.4%)	5 (17.2%)	6 (8.5%)	0.027
Intraoperative drain placement	36 (85.7%)	22 (75.9%)	58 (81.7%)	0.290
Peri-cholecystic fluid collection	9 (21.4%)	7 (24.1%)	16 (22.5%)	0.936
Presence of bile leak	7 (16.7%)	3 (10.3%)	10 (14.1%)	0.312
Previous history of biliary inflammation (cholecystitis or cholangitis)	13 (30.95%)	9 (31.0%)	22 (30.9%)	0.844
Other Complication				0.091
Adhesions/empyema	1 (2.4%)	0 (0.0%)	1 (1.4%)	
Cholecystoenteric fistula	0 (0.0%)	2 (6.9%)	2 (2.8%)	
Liver injury/bleeding	0 (0.0%)	3 (10.3%)	3 (4.2%)	
None	34 (80.95%)	23 (79.3%)	57 (80.3%)	
Perforation	1 (2.4%)	0 (0.0%)	1 (1.4%)	
Port-site iatrogenic bowel injury	1 (2.4%)	0 (0.0%)	1 (1.4%)	

LSTC: laparoscopic subtotal cholecystectomy; OTC: open total cholecystectomy; SD: standard deviation; BDI: bile duct injury.

## Data Availability

The data is available from the corresponding author upon reasonable request.
